# End-to-End AI-Based Point-of-Care Diagnosis System for Classifying Respiratory Illnesses and Early Detection of COVID-19: A Theoretical Framework

**DOI:** 10.3389/fmed.2021.585578

**Published:** 2021-03-31

**Authors:** Abdelkader Nasreddine Belkacem, Sofia Ouhbi, Abderrahmane Lakas, Elhadj Benkhelifa, Chao Chen

**Affiliations:** ^1^Department of Computer and Network Engineering, College of Information Technology, UAE University, Al Ain, United Arab Emirates; ^2^Department of Computer Science and Software Engineering, College of Information Technology, UAE University, Al Ain, United Arab Emirates; ^3^Cloud Computing and Applications Research Lab, Staffordshire University, Stoke-on-Trent, United Kingdom; ^4^Key Laboratory of Complex System Control Theory and Application, Tianjin University of Technology, Tianjin, China

**Keywords:** COVID-19, intelligent learning, respiratory illness, health diagnosis, e-health

## Abstract

Respiratory symptoms can be caused by different underlying conditions, and are often caused by viral infections, such as Influenza-like illnesses or other emerging viruses like the Coronavirus. These respiratory viruses, often, have common symptoms: coughing, high temperature, congested nose, and difficulty breathing. However, early diagnosis of the type of the virus, can be crucial, especially in cases, such as the COVID-19 pandemic. Among the factors that contributed to the spread of the COVID-19 pandemic were the late diagnosis or misinterpretation of COVID-19 symptoms as regular flu-like symptoms. Research has shown that one of the possible differentiators of the underlying causes of different respiratory diseases could be the cough sound, which comes in different types and forms. A reliable lab-free tool for early and accurate diagnosis, which can differentiate between different respiratory diseases is therefore very much needed, particularly during the current pandemic. This concept paper discusses a medical hypothesis of an end-to-end portable system that can record data from patients with symptoms, including coughs (voluntary or involuntary) and translate them into health data for diagnosis, and with the aid of machine learning, classify them into different respiratory illnesses, including COVID-19. With the ongoing efforts to stop the spread of the COVID-19 disease everywhere today, and against similar diseases in the future, our proposed low cost and user-friendly theoretical solution could play an important part in the early diagnosis.

## 1. Introduction

People usually take breathing and respiratory health for granted and often forget that their lungs are vital organs, which are vulnerable to infections and injury. Respiratory diseases are, according to the World Health Organization (WHO), among the leading causes of disability and death in the world (1). Respiratory diseases include “acute respiratory infections as well as chronic respiratory diseases, such as asthma, chronic obstructive pulmonary disease, and lung cancer” (2). Multiple factors can aggravate respiratory conditions, such as: tobacco smoke exposure either direct or indirect; heavy exposure to air pollution; occupational related disorders; malnutrition and low birth weight, but most commonly by exposure to viruses, such as the influenza virus or the Coronavirus (3). Making a timely and accurate diagnosis is essential for treatment as symptoms of respiratory illnesses are often very similar to each other (4), which can cause confusions that can lead to misdiagnosis. This may result in catastrophic consequences of further spread of the infection, particularly during pandemics, such as COVID-19 pandemic. Therefore, ensuring a diagnostic differentiator is highly crucial for timely and accurate prognosis and appropriate actions (5).

Cough is considered a key symptom of respiratory diseases (6). Cough is considered a key pulmonary disease symptom and a natural defense mechanism of the human body to protect the respiratory system (7). In normal conditions, a cough is a result of a contraction of respiratory muscles which compress the air in the lungs. This contraction follows the glottis closure after an inspiration of air (8) and occurs immediately before the sudden glottis reopening, which leads to a rapid air expulsion from the lungs to clear breathing passages. Cough properties are unique and repeatable for a given subject (9). A cough can be either productive or non-productive (10). A productive cough produces phlegm or mucus, clearing it from the lungs, while a non-productive cough, also known as a dry cough, does not produce phlegm or mucus. Analyzing the cough sound during therapy can give relevant information about the coughing pathophysiological mechanisms that result in specific cough patterns (11). Information on the glottis behavior in different respiratory conditions could also be retrieved from the cough sound (11). Change in cough sound is considered a critical indicator of the respiratory disease progress and the effectiveness of a therapy (11).

Characteristics of voluntary and involuntary (i.e., spontaneous or reflex) coughs have increasingly been analyzed to detect and characterize lung disease (12). Automated real-time and reliable lab-free tools for cough characterization and classification could be valuable for timely and accurate diagnosis and differentiating between different respiratory illnesses, which is crucial for correct treatment (13). This can be particularly useful in parts of the world with limited access to laboratory resources (14). Coughs are often seasonal events, therefore a cough classifier or detector shall have an extremely low false alarm rate to be considered clinically reliable. Moreover, this system shall be highly sensitive to changes in cough sounds to detect any infrequent event (15). To the best of our knowledge, there is no standard approach to evaluate cough sounds automatically that has been implemented, despite the fact that several approaches have been proposed in literature (11, 16). We also recognize that to achieve accurate diagnosis, data of other accompanying symptoms, such as temperature, must be used in conjunction with the cough data.

**Figure 1:**
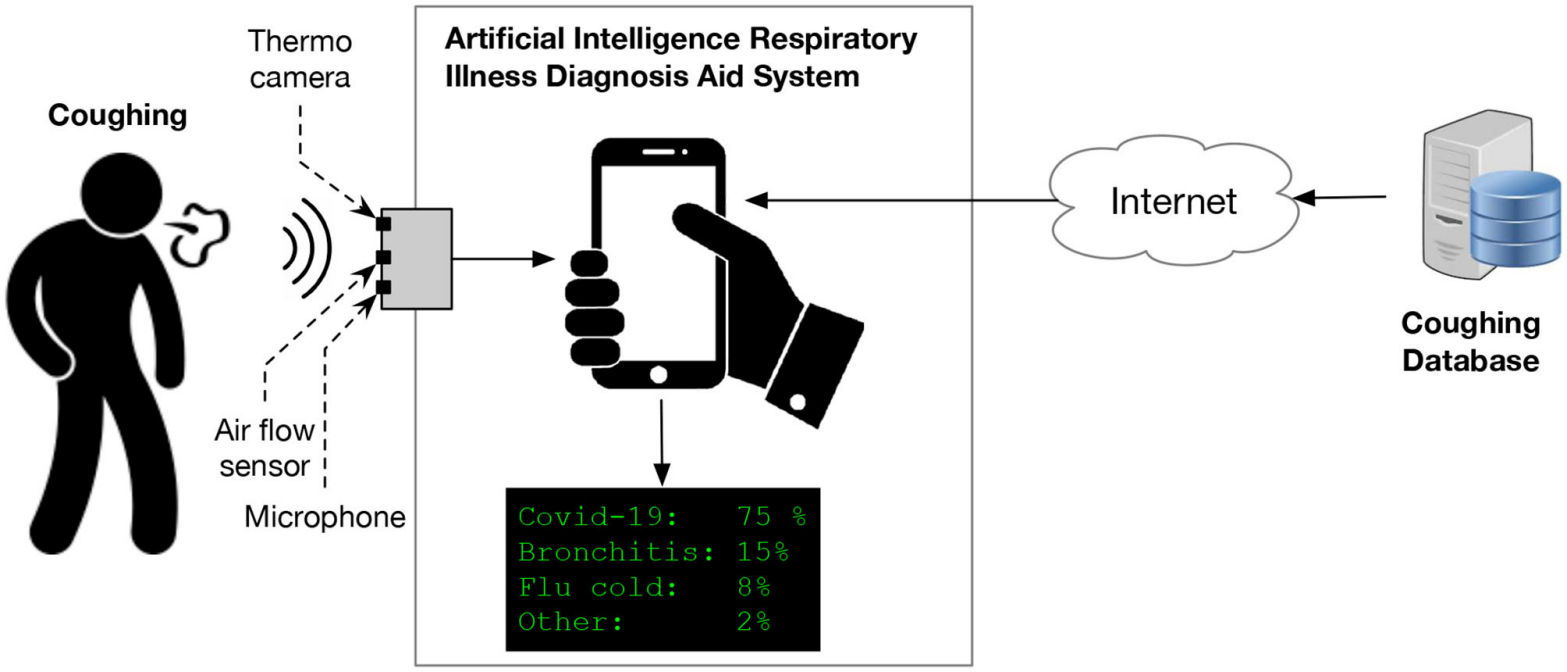
Illustration of the proposed AI-based diagnosis aid system for simultaneously recording, detecting, and classifying cough characteristics using fuzzy logic methods.

This paper proposes a theoretical end-to-end point-of-care system, supported by artificial intelligence (AI) module for classifying and diagnosing different respiratory illnesses, including early detection of COVID-19. The novel proposed theoretical system is composed of hardware and software components. The system will be able to record patients' or users' symptoms, including body temperature, cough sound, and airflow, using sensors. The recorded data will then be translated to health data, which will be processed by a machine learning module, to find patters and classify the combined symptoms for different respiratory conditions, including COVID-19. A customized mobile application (app) will be developed and used for data processing and visualization. The app will allow the users to interact with the system's parameters, including the option to submit their results to physicians electronically. Patients' data will be stored securely in the cloud. Figure 1 provides a high level illustration of the proposed system. The remainder of this theoretical framework paper is as follows: section 2 presents related works identified in the literature. In section 3, we explain the main components of the proposed system and its detailed architecture. In section 4, we conclude by discussing the prospective of the proposed framework, the challenges and the limitations.

## 2. Related Work

Several studies have been conducted to classify and detect lung-related diseases using AI. Liu et al. (17) proposed a classification algorithm of lung sounds based on multilayer perceptron network and Hilbert-Huang transform features for non-invasive diagnosis of pulmonary diseases. The algorithm was tested using the R.A.L.E. database with a multi-layer perceptron classifier and achieved an averaged classification accuracy of 95.84%. Aykanat et al. (18) proposed a non-invasive classification method of recorded respiratory sounds using an electronic stethoscope. They recorded with this device 17,930 lung sounds from 1,630 subjects. Their results showed that by using convolutional neural network (CNN) and support vector machine (SVM), they could accurately classify respiratory sounds. Azam et al. (19) presented a scheme to detect respiratory patterns that are irregular due to respiratory diseases. They used 255 breath cycles captured using a smartphone under natural setting. Their experiments showed an accuracy around 75% using SVM for asthmatic inspiratory cycles and complete respiratory sounds.

Few AI systems have been proposed and/or developed to detect respiratory illnesses (20). Among them, *FluSense* (21), which is a contactless platform for syndromic surveillance of influenza-like illness used in waiting areas of hospitals. *FluSense* captures bio-clinical signals related to physical symptoms of influenza-like illness of individuals waiting in hospitals in privacy-sensitive and an unobtrusive manner. *FluSense* uses a thermal camera, a microphone array and a neural computing engine to characterize cough sound changes of individuals waiting in hospitals in a real-time manner. The researchers conducted a 7-month study in four public waiting areas equipped with *FluSense* within the hospital of a large university from December 2018 to July 2019 (21). In that study, *FluSense* collected and analyzed 21 million non-speech audio samples and around 350,000 waiting room thermal images. The study (21) showed that *FluSense* accurately predicted the patient daily counts (Pearson correlation coefficient = 0.95). The *FluSense* platform did not take into consideration all respiratory illnesses nor additional health data. Cough data is important and relevant features but not sufficient one to be used for all respiratory illnesses.

With the recent rise in the new Coronavirus pandemic, early and accurate testing has been crucial due to the fast spreading feature of COVID-19, which caused more than 113 million confirmed cases and more than 2.5 million deaths up to late Feb 2021 (22). Among the important factors for the spread of COVID-19 have been lack of testing and erroneous diagnosis, which might be due to inaccurate testing or confusion with flu-like symptoms (23). Two main mechanisms to detect the Coronavirus disease were adopted at the beginning of the pandemic (24): (i) clinical scan images analysis of chest computed tomography (CT), and (ii) results of blood tests. COVID-19 patients, commonly, manifest persistent fever, tiredness, and dry cough. Although little peer-reviewed research existed on the diagnosis of COVID-19 during the first months of its spread worldwide, there has been a rapid response in the research community toward COVID-19 diagnoses and prediction using AI-based software on medical imaging (25–27). Chen et al. (28) have developed an engine to detect COVID-19 disease using high resolution CT images and deep learning. It has been deduced in Wynants et al. (27) that the prediction models for COVID-19 diagnosis are not well-reported with a high risk of bias. There is therefore a need to combine these models with other diagnostic methods, such as lab tests. However, these methods are costly and time consuming, which was problematic for many countries which do not have the capacity to accommodate tests for large populations. Cheaper rapid tests have emerged recently to increase the testing capacity of many countries (29). However, these low-cost and rapid tests are often non-trusted, with many cases of false positives or false negatives. For this reason, several researchers have started looking into alternative solutions to detect COVID-19.

Some researchers have proposed a novel framework on how to detect COVID-19 using on-board smartphone sensors (30). However, their idea is still in the conceptualization phase and has not yet been implemented. The proposed solution is designed for certain types of smartphones, which may not be available and affordable to a large number of people. In addition their proposed framework is designed for COVID-19 diagnosis only. Imran et al. (31) have proposed *AI4COVID-19*, an AI-based app, that can diagnose and distinguish COVID-19 coughs from other non-COVID-19 coughs, such as pertussis and bronchitis. A similar tool has been proposed by Faezipour and Abuzneid (32), who have highlighted the benefits of developing a self-testing app using breathing sounds for COVID-19 diagnosis. They suggested that using an AI-based app using solely breathing sounds could estimate the user's lung volume and oxygenation, and diagnose healthy and unhealthy cases, including COVID-19. Laguarta et al. (33) have discriminated the asymptomatic COVID-19 infections through cellphone-recorded coughs using four biomarker patterns—vocal cord strength, sentiment, lung and respiratory performance, and muscular degradation—that are specific to COVID-19. Brown et al. (34) have analyzed a large-scale crowdsourced dataset of respiratory sounds that were collected to assist in diagnosis of COVID-19. They have concluded that it is possible to distinguish COVID-19 cough sounds from healthy cough using a simple binary machine learning classifier. This points to the potential of using cough sounds to diagnose COVID-19 (35–37). The cough sound can also be used to detect other respiratory illnesses. Moschovis et al. (38) used a smartphone app, *SMARTCOUGH-C 2*, to collect and analyze cough sounds to detect respiratory diseases in children. Analyzing the sound cough to classify respiratory disorders other than COVID-19 has also been investigated by Taquee and Bhateja (39). To the best of our knowledge, there is no end-to-end solution proposed to diagnose COVID-19 and other respiratory disorders using cough sound in combination with other health data. Hence the need for our contribution. The aim of this paper is to develop an end-to-end point-of-care diagnosis devise, that can collect data of combined common symptoms in respiratory illnesses (e.g., cough, body temperature, and airflow) and classify them as a diagnostic differentiator, detecting even asymptomatic COVID-19. The system has also the potential to be adaptable to early diagnosis of newly emerging respiratory illnesses.

## 3. The Proposed System

The objective of cough classification is to develop an automatic system that is capable of classifying various attributes of coughs, such as the intensity of the cough, time-frequency, energy distributed, or whether the cough is wet or dry. Different respiratory diseases, such as Bronchitis, Tuberculosis, Asthma, may have different effects on the pulmonary system, and therefore, are identifiable through the changes observed on the cough sound. For instance, coughs from asthmatic patients tend to have different energy signatures than that from non-asthmatic patients. In particular, asthmatic coughs exhibit more energy in the low frequency (40). On the other hand, the study of the cough phases reveals that dry coughs have lower intensity than wet coughs in phase two. In addition, during this phase, most of the signal intensity of the wet coughs is found to be within 0–750 Hz range, whereas that of the dry coughs is within 1,500–2,250 Hz range (41). Thus, most of cough recording experiments have been using a sampling frequency between 48,000 and 22,050 Hz to cover all cough types. Before designing the architecture of proposed system, it is important to first understand the cough audio preprocessing phase, which is described below.

### 3.1. Cough Audio Preprocessing

#### 3.1.1. Cough Audio Analysis

**Figure 2:**
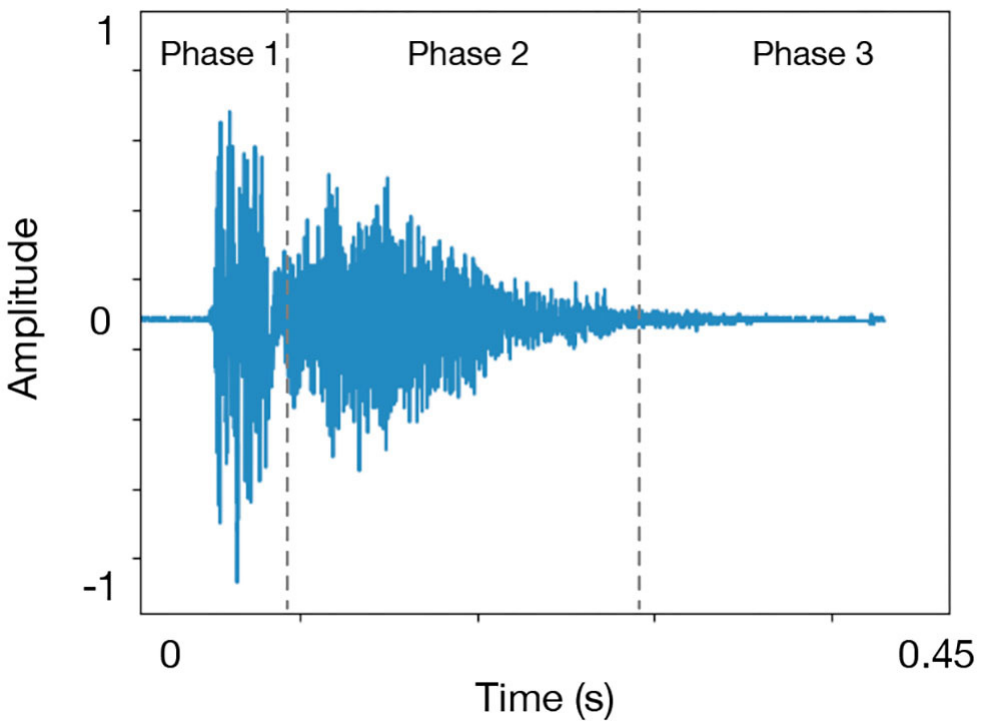
A typical cough sound structure. There are three common patterns of cough based on the number of phase, such as three-phase cough, two-phase cough, and peal cough.

The acoustic sound of a cough is generated by the contractions of the respiratory muscles. The cough, with its typical sound, is the result of the sudden opening of the glottis opening suddenly due to a rapid exhalation of air from the lungs. A typical cough sound signal consists of three phases as shown in Figure 2:

1. a rapid explosive phase which is recognized by an initial burst of emerging sound yielding a high frequency due to the vibrations resulting from the air flowing in the narrow bronchial airways.
2. an intermediary phase which decays as air is flowing in a steady-state while the glottis is fully open. This phase gives the duration to the whole cough. When the sputum is present a higher frequency component may be added to the signal.
3. a phase named voiced and that is not present in most coughs. This phase is characterized by a narrowing of the glottis again leading the vocal cords to get close to each other.

The first two phases are ubiquitous across all coughs and will be useful in determining the start and end of a cough using an energy-based criteria. The majority of the coughs duration is around 400 ms (Here we chose 50 ms) (42). For instance a dry cough is characterized by the absence of any mucus or sputum (10). That is, all the three phases are visible in a dry cough signal. Initially, a burst of high energy is observed followed by less energy in the second phase at higher frequencies. However in the case of a wet cough sound signal more energy is observed in Phase 2 at higher frequencies. Typically, a wet cough, symptomatic of bronchitis, asthma, and pneumonia, is produced by inflammation and secretion of mucus and sputum in the lower airways caused by either a bacteria or a virus (10).

#### 3.1.2. Cough Segmentation and Detection

**Figure 3:**
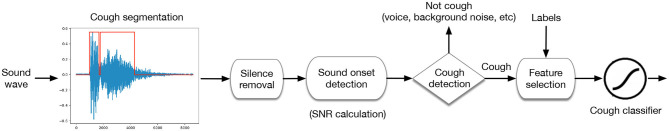
Workflow for cough audio processing. For segmentation phase, predefined thresholds can be chosen based on the physiology of cough sounds. The raw recorded sounds contains a lot of silent fragments (with low intensity) and background noise. Therefore, silence removal phase is required for saving storage space. For signal quality check, an estimate of the signal-to-noise ratio (SNR) can be computed by taking the ratio of the power of the cough part of the signal to the ratio of the rest of the signal.

One of the first tasks of cough audio analysis is to be able to detect and identify a cough signal then classify it using some biomarkers (see Figure 3). Several research studies have addressed cough detection using different methods. For instance, in Barry et al. (43), the authors used Linear Predictive Coding and Mel-Frequency Cepstral Coefficients to model the sound of coughs, and used a Probabilistic Neural Network to classify time windows as containing or not a cough. Other researchers have used Hidden Markov Model (HMM) with Mel-Frequency Cepstral Coefficients (MFCCs) to be fed as input (44).

This process consists of cleaning the cough sounds dataset by filtering out all the interferences and the environmental noise from the audio frames and keeping only the relevant frames to the cough. of audio separation consists of extracting the cough sounds. There are several methods for source separation including Independent Component Analysis (ICA) (45), Blind Source Separation (BSS) (46), and Informed Source Separation (ISS) (47).

#### 3.1.3. Feature Selection and Discriminant Analysis

**Figure 4:**
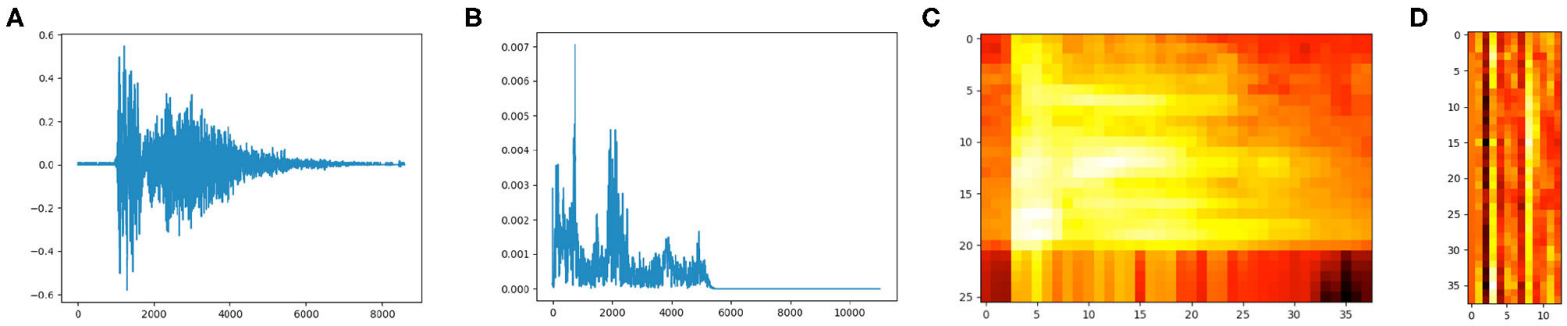
Cough time-frequency representation analyses. **(A)** Cough signal 16-bit, frequency range 22050Hz. **(B)** Power spectrum of the cough audio frame (FFT). **(C)** Spectrogram. **(D)** MFCC.

Feature extraction is an important step toward classification of cough sounds. However, creating a classification model with from a dataset with high-dimensionality is time consuming and may converge to a local minima given the large search space. Therefore, selecting a reduced set of relevant features in an audio sample can improve immensely the performance generating a classification model (48). There are many techniques for feature selection including Shannon Entropy (SH), Fisher score, Mel-Frequency Cepstral Coefficients, and Zero Crossing Rate (ZCR). However, MFCC method has gained popularity due to its efficiency in the analysis of speech and sound signals in general, and is therefore opted for in the analysis of cough sounds. Features selection during the pre-processing of cough sounds is 2-fold: first, reducing the dimensionality for the feature matrix classification, and second, extracting the most dominant information present in the cough sound. Prior to feature extraction, any noise must be filtered out of the cough sound. The quality and performance of the classification process depends strongly on how well the process of features extraction has been done (see Figure 4).

#### 3.1.4. Mel-Frequency Cepstral Coefficients

MFCCs are commonly used in audio processing for sound pattern recognition. In this method assumes that a sound is produced during the passage of small glottal pulses through vocal channel filter. Therefore, it only makes sense to exploit a small set of features in the signal. That is, for MFCCs, usually about 10–20 are enough to represent the overall spectral envelope of the cough signal. MFCC analysis shows the power spectrum of the signal, and is often used to describe its timber. MFCCs are obtained after putting the signal spectrum on a non-linear Mel-scale of frequency, then obtaining the log-power spectrum from the result and finally applying a cosine transform. A cough sound has a complex signal structure carrying critical information that can contribute to he discrimination between various phases of the signal. The use of Mel scale is motivated by the fact that the features of an audio signal are better discerned by a human ear at low frequencies than they are at high frequencies. Therefore, Mel scale ensures that signal features match more closely what humans hear. The Mel scale is obtained using the following conversion formula:

(1) $$\begin{array}{l} M\left(f\right)=1125\ln\left(1+\frac{f}{700}\right) \end{array}$$

(2) $$\begin{array}{l} M^{-1}\left(m\right)=700\left(\text{exp}\frac{m}{1125}\right)-1 \end{array}$$

MFCCs calculated following few steps starting by framing the signal into shorter frames (20–40 ms frames). That is, for a signal sampled at *f*_*s*_ Hz, and a standard 25 ms, the frame length is equal to 0.0025 × *f*_*s*_ samples. For each sample, the periodogram estimate of the power spectrum is calculated. The Mel filter bank is then applied to the power spectra and the sum of the energy in each filter is calculated. The next step consists of calculating the logarithm of all the filter bank energies, and the discrete cosine transform (DCT) of the result. From the computed DCT, only coefficients 2–13 are retained as Mel coefficients.

#### 3.1.5. Cough Classification and Machine Learning

Classification of cough sounds is a helpful tool for identifying the underlying cause of coughs. Several methods for automatic cough classification have been developed to identify various cough types and the thus the pulmonary disease. The most common used classifiers are Gradient boosted decision trees (XGBoost), Deep Neural Network (DNN), Convolutional Neural Network (CNN), Recurrent Neural Network (RNN), and Fuzzy Deep Neural Network (FDNN).

### 3.2. Proposed System Architecture

Does cough sound contain sufficient information to be used for distinguishing among all respiratory illnesses? Is it possible to detect and classify COVID-19 infection through cough sounds using artificial intelligence algorithms? In this concept paper, a medical hypothesis of classifying respiratory illnesses using cough data from a forced-cough device recording is discussed with some proposed explanations made on the basis of limited evidence as a starting point for further investigation. We propose a theoretical design of reliable user-friendly AI based system for early detection of COVID-19 and other respiratory illnesses. This integrated hardware-software system will have two main components:

1. A novel hardware composed of several sensors (e.g., microphone, and thermal imaging tool, and a cough sound-recording device),
2. An AI software for cough classification and flu type recognition.

**Figure 5:**
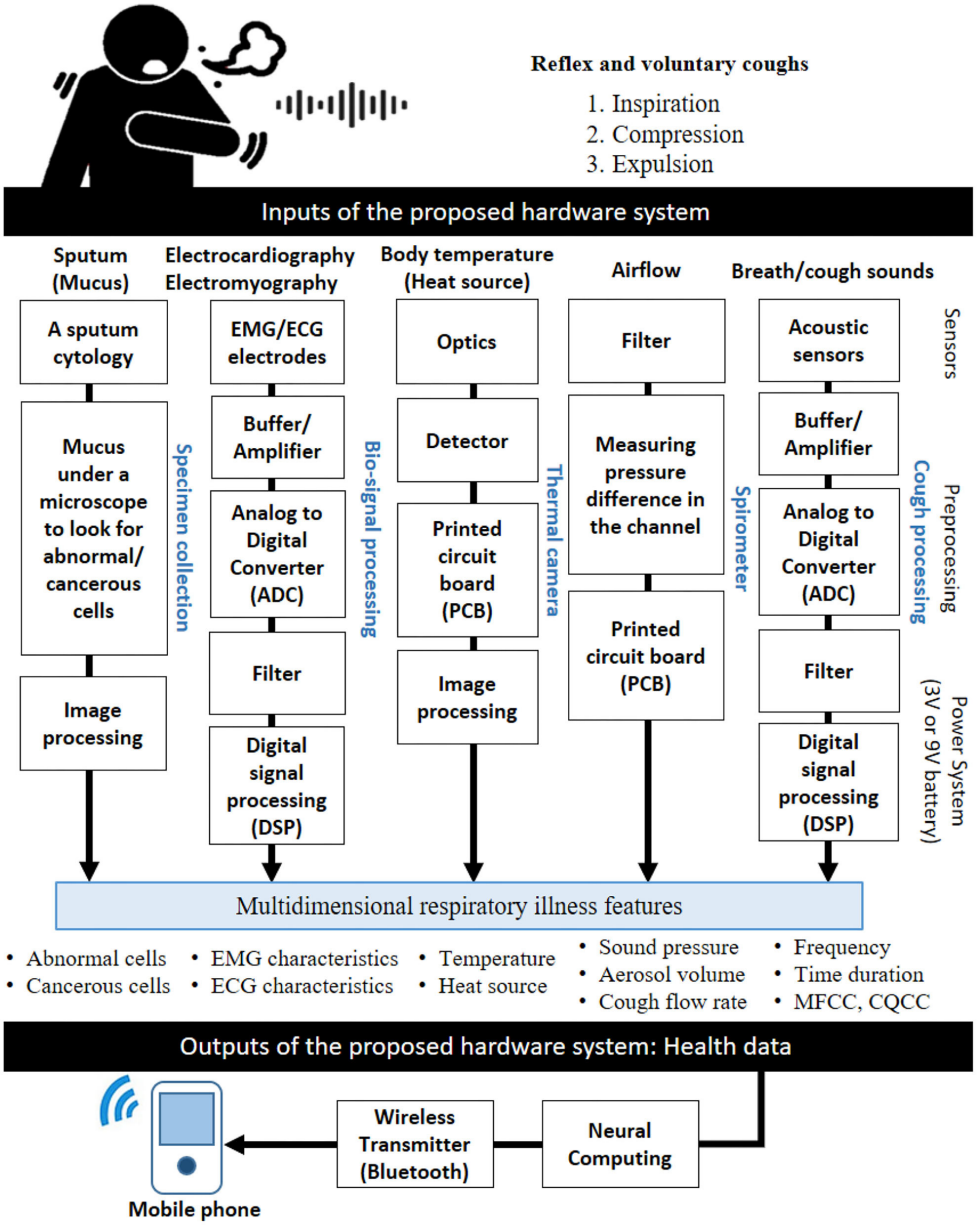
Hardware architecture of our proposed low-cost system to collect health data. The hardware contains relevant sensors to measure some parameters, such as cough sound, breathing, body temperature, and so on to be translated to relevant health data (e.g., audio signal features commonly used in cough classification). Several sensors are needed to get relevant information from the human body (e.g., thermal camera, acoustic sensor, accelerometer, portable piezo contact microphone, airflow sensor, and EMG/ECG electrodes).

Figure 5 shows all necessary hardware components to collect health data from healthy and unhealthy participants. In the following paragraphs, we will explain each component in details.

#### 3.2.1. Collecting Data

**Figure 6:**
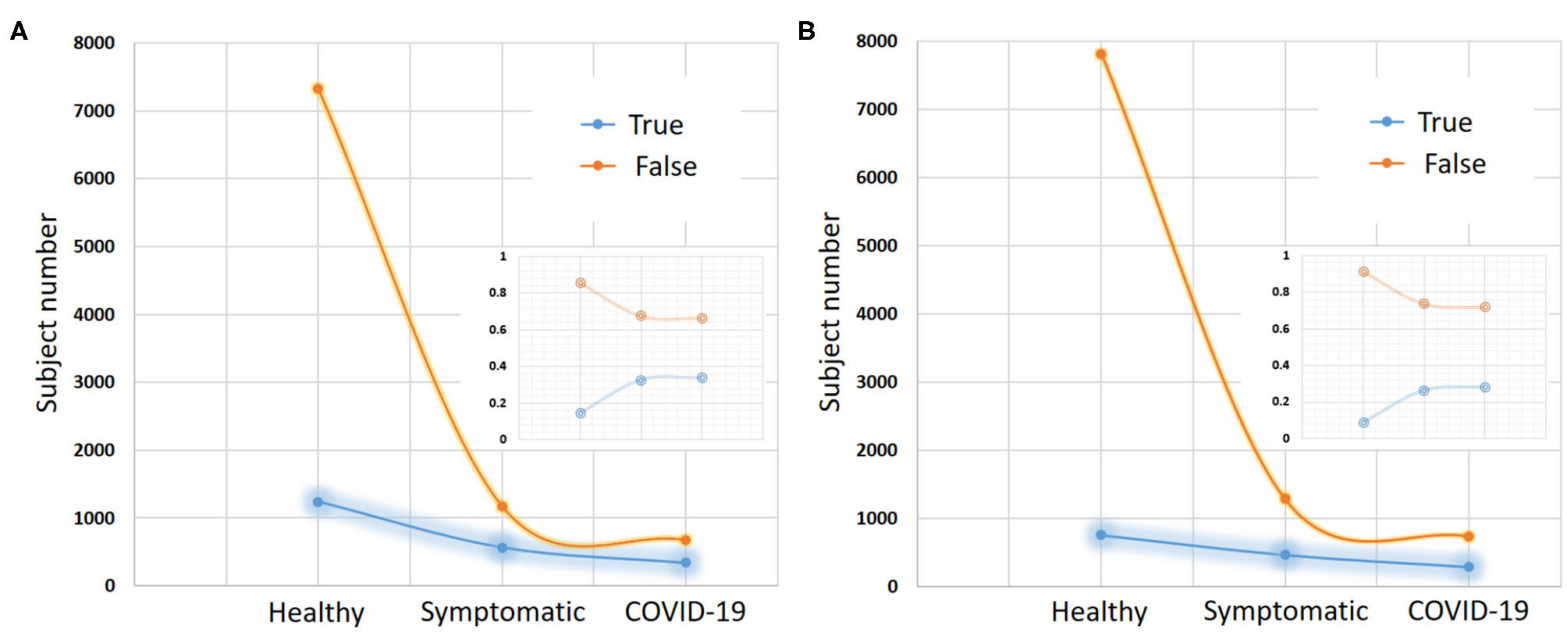
COUGHVID recording results. This cough dataset-based public recording platform provides over 20,000 crowd-sourced cough recordings representing a wide range of subject ages, genders, geographic locations, and COVID-19 statuses. **(A)** Shows the presence or absence of any respiratory condition of each subject. **(B)** Shows the presence or absence of fever/muscle pain for each subject.The gap between true and false decreases within two statuses: symptomatic and COVID-19 subjects compared to healthy subjects in both respiratory condition and fever muscle pain. The small graph in each figure represents a normalization of the number of recorded data for each status.

Collecting data strategy is one of critical phases to build a trusted diagnosis model. The data can be collected using specific sensors for each input. For example, collecting cough sound requires a simple microphone for recording the sound via smartphone app or a web browser (49, 50). However, it is still a challenge to record big data for each respiratory illness. One way is to kindly ask people over the world to volunteer their cough sounds via online platforms. We may also collect basic demographics, medical history, and a few seconds of reflex and voluntary coughing samples. The participants should ensure that recording the sounds was in a quiet environment to avoid noisy sounds. The participant's anonymity and privacy are protected and no personal information is collected. If agreed on by the participant, the location may be collected as location information is particularly useful to draw a map of respiratory illnesses. The collected data may be made available as open source to be used for promoting science and fighting respiratory diseases. Therefore, a process of ethical approval and clear informed consent from the participants may be required before and during the collection. Table 1 shows recent online cough recording platforms. For developing an trusted AI model-based respiratory illnesses' diagnosis, many information can be recorded as inputs, such as cough sound, body temperature, airflow, EMG, ECG, and mucus. The COVID-19 pandemic has proved that it is time for everyone to contribute to public-cough datasets development which can include all cough types for each respiratory illness. The action can get sped up by encouraging patients, hospitals, and healthcare companies for donating their medical records to science. Figure 6 shows an example of large public cough database [COUGHVID (49), the Embedded Systems Laboratory (ESL) at École polytechnique fédérale de Lausanne (EPFL), Switzerland].

**Table 1:** A list of some online recording platform-based cough databases.

**Dataset**	**Source**	**Recording platform**	**Recording type**	**References**
Cambridge database	University of Cambridge, UK	https://www.covid-19-sounds.org/	Short recordings of cough and breathing and report symptoms (healthy and non-healthy participants).	(34)
MIT database	Massachusetts Institute of Technology (MIT), USA	https://opensigma.mit.edu/	Cough sounds from healthy and COVID-19 subjects, including asymptomatics.	(33)
NYU database	New York University (NYU), USA	https://www.breatheforscience.com/	Studying the link between respiratory diseases and breathing patterns in the US population.	(51)
Virufy	Stanford COVID-19 Response Innovation Lab, USA	https://virufy.org/data	Virufy is a global applicability of crowdsourced and clinical datasets for AI detection of COVID-19 from cough.	(52)
NOCOCODA	Carleton University's Institutional Repository, USA	From online interviews with COVID-19 patients (e.g., published interviews on social media and YouTube videos)	This coronavirus coughs database contains cough events obtained from online interviews with COVID-19 positive individuals.	(50)
COUGHVID	École Polytechnique Fédérale de Lausanne (EPFL), Switzerland	https://coughvid.epfl.ch/	Cough sounds from healthy, symptomatic, and COVID-19 participants.	(49)

#### 3.2.2. Cough Sound Recording

**Figure 7:**
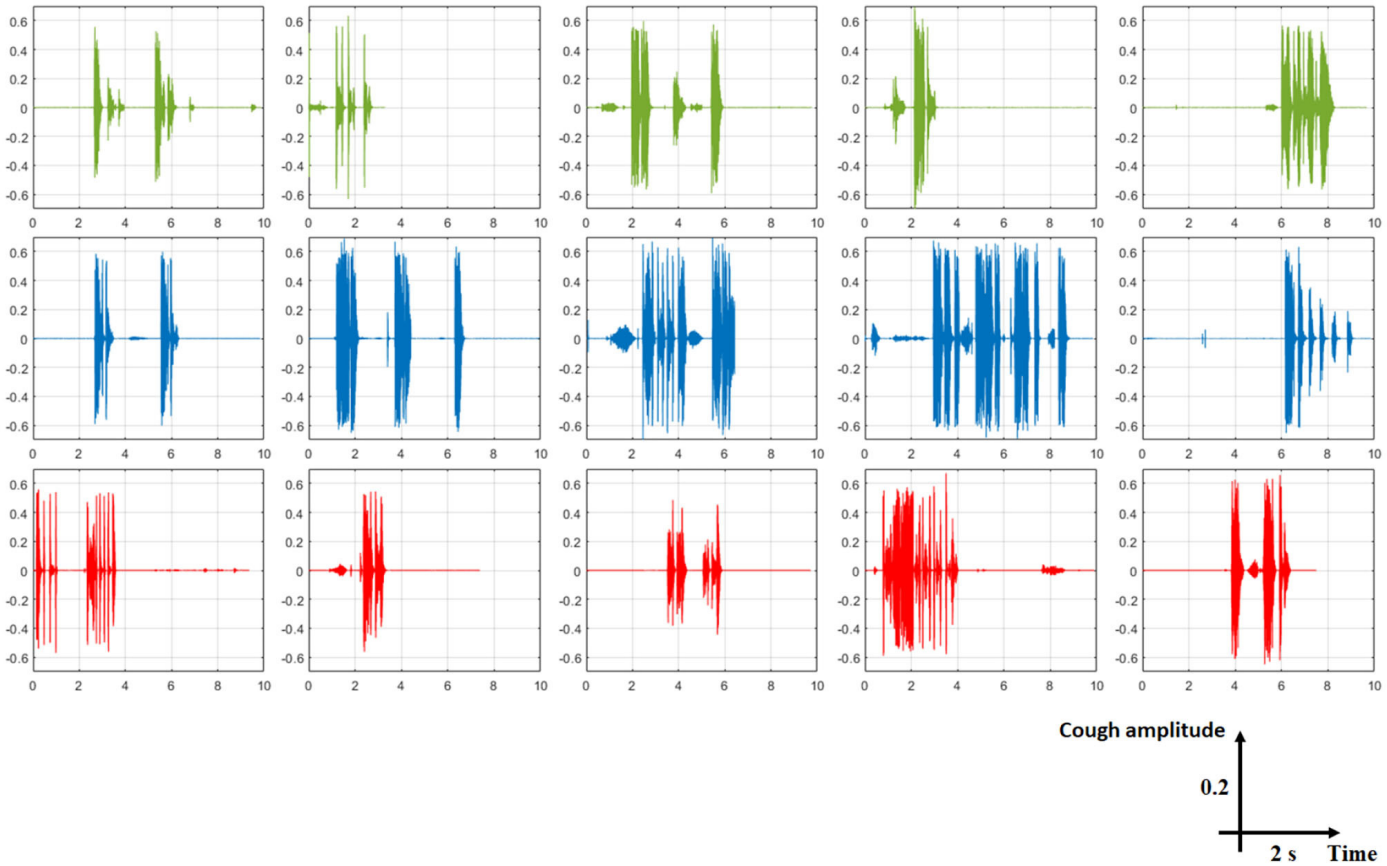
Representative examples of some cough types from COUGHVID database. Each signal has a total duration of 10 s. The five first signals in green color are from healthy subjects, the blue ones are from symptomatic subjects, and the red ones are from COVID-19 subjects. From this figure, it is clear that detecting cough sounds, to be distinguished from noise or speech, is an important step before classifying expert-labeled cough types.

The proposed system architecture is designed in order to extract useful clinical information from many inputs, such as cough sounds. For recording audio of people coughing, breathing (which indicate labored or irregular breathing) or even talking, we need a microphone to convert acoustical energy (sound waves) into electrical energy (the audio signal). Then, we need amplifier, analog to digital converter (ADC), and digital signal processing (DSP) for prepossessing phase. Cough characteristics and its acoustic features depend on the velocity of airflow, dimensions of the vocal tract and airways, and location of sound generated. Amplitude of the cough sound, intensity, duration, frequency, time-frequency representation (spectrogram which is a visual representation of the spectrum of frequencies of a signal as it varies with time), the mel frequency cepstral coefficients, the constant-Q cepstral coefficients (CQCC), and so on can be used for feature extraction phase as cough sound pattern. However, if the cough sound was not recorded in quiet room then blind source separation and independent component analysis can be used to find the right signal. Figure 7 shows signals of three cough types (healthy, symptomatic, and COVID-19). Some researchers have already started using cough database for classifying some cough statues, such as healthy, symptomatic, asymptomatic, and COVID-19 coughs (33, 53).

#### 3.2.3. Spirometer

A spirometer is an apparatus for measuring the volume of air inspired and expired by the lungs of healthy or unhealthy participants. It measures ventilation, the movement of airflow into and out of the lungs which a high indicator for early diagnosis of any respiratory illnesses. For this subsystem, the input is airflow and the outputs could sound pressure, aerosol volume, and cough flow rate.

#### 3.2.4. Thermal Camera

The infrared thermal imaging camera can be used for detecting elevated body temperatures which may indicate the presence of a fever, a symptom of many respiratory illnesses, such as COVID-19. Detecting people with a potential fever may contain or limit the spread of many contagious respiratory illnesses through identification of infected individuals showing fever symptoms. Thermal cameras are passive devices that don't emit any radiation, rather they use infrared radiation emitted form objects (human body in this case) to deliver high-resolution images without the need of any additional illumination. These cameras provide a visual map of skin temperatures in real time. They allow the operator of a critical public infrastructure (e.g., airports, train stations, and schools) to non-invasively scan the crowd or individuals to avoid the spread of some infectious disease.

#### 3.2.5. EMG/ECG Processing

COVID-19 and many respiratory illnesses may cause trouble breathing, liver problems or damage, heart problems, and kidney damage. In most cases, the lungs might become inflamed, making it tough for patients to breathe. This can lead to pneumonia, an infection of the tiny air sacs (called alveoli) inside the lungs where the blood exchanges oxygen and carbon dioxide. However, during respiratory biofeedback, we can place some sensors or electrodes around the abdomen and chest to monitor the breathing patterns and respiration rate. Electrocardiogram (ECG) can be used for measuring the heart rate and how your heart rate varies (e.i., atrial and ventricular depolarization and repolarization are represented on the ECG as a series of waves PQRST: the P-wave, the QRS complex, and the T wave). In addition, electromyogram (EMG) can be also used for monitoring the electrical activity that causes muscle contraction around heart or chest wall movements or even for getting cough sound from the throat.

#### 3.2.6. Sputum Cytology

A sputum cytology is used for testing lung secretions or phlegm to look if there are some cancerous cells. The patient coughs up a sample of sputum (mucus), which is checked under the microscope to identify possible cancer cells or determine whether abnormal cells are present. However, using automated sputum cytometry, such as LungSign test for lung cancer, lung ultrasound for pneumothorax, or optical automation for sputum cytology can be also useful compared to the conventional cytology.

#### 3.2.7. Artificial Intelligence Based Algorithm

**Figure 8:**
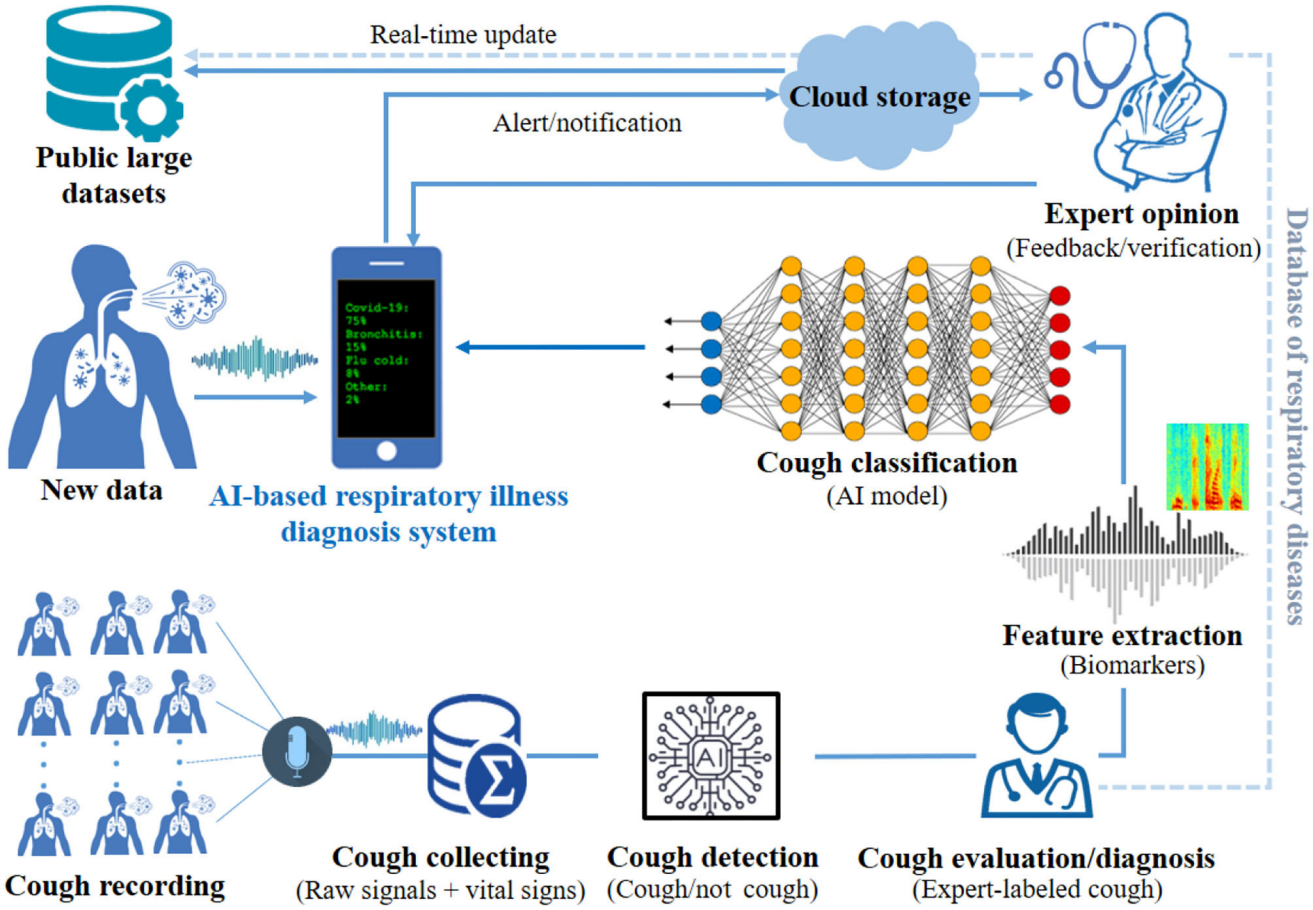
Software design of the proposed AI-based cough diagnosis system. This system includes all phases with the possibility of sharing the collected stored database and the preliminary diagnosis results with researchers/medical doctors. The sharing approach of the database will lead to develop more robust open-source algorithms to be used for detecting and classifying any respiratory illnesses.

After extraction some relevant patterns from system inputs and building a feature vector or matrix, we will give the data to some machine learning or deep learning methods [e.g., support vector machine (SVM), artificial neural network (ANN), convolutional neural network (CNN)]. However, machine learning algorithms almost always require structured data, while deep learning networks rely on layers of ANN. Figure 8 shows cough diagnosis-based software design from collecting data until sending the health data with the preliminary diagnosis to the patients and/or physicians. This proposed software design can used for all relevant collected health data, such as breathing instead of coughing inputs. The proposed software design has two phases: offline and online. For offline analysis, all collected data will be used for building a classification model by dividing the data into 80–90% training data and 20–10% test data. For classification method, deep learning is the best choice for big raw data. However, the number of class of respiratory illnesses is very high. Therefore, fuzzy logic might be an option to give a percentage of truth of each class. The model will be made based on many participants' data to be able to generalize it later in the testing phase. In addition, normalization of the data is also important. After fixing all parameters of the supervised classifier and building the model, we will test it on new data to check its performance. For real-time analysis, an online classification algorithm based on offline model (or dynamic model) is needed. The classification result of any respiratory illnesses will be display for the user on the smartphone and it can be sent to experts via cloud service. Further details on the implementation of different machine learning algorithms for cough classification is presented in the subsequent sections.

**Figure 9:**
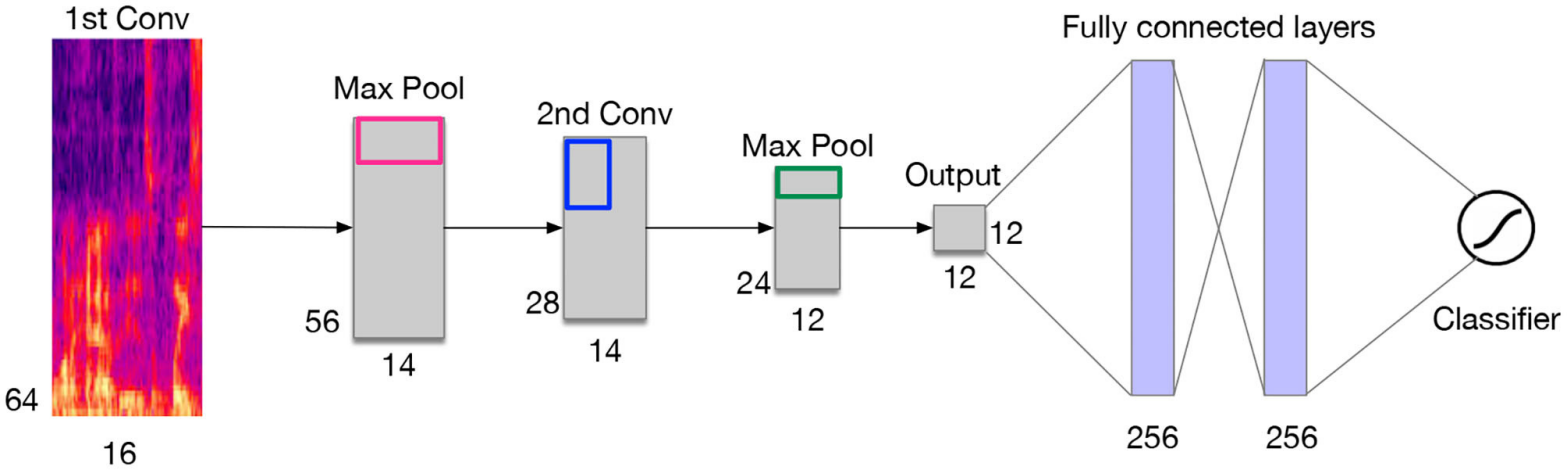
Example of a CNN architecture for a cough sound classifier.

1. Deep neural networks are feed-forward networks that are characterized by an input layer and an output layer and multiple hidden layers, through which information is typically processed by a logistic function. The total input at a given layer is mapped and sent forward to the next layer. In the case of signal classification, the audio input, represented by its MFCCs obtained from the raw waveform, is computed at each layer by concatenating the MFCCs. MFCCs are better adapted to facilitate discrimination with Hidden Markov Model (HMM) that are used in this context to capture the temporal information of the audio signals. DNNs also requires a fine-tuning step, which is a back propagation tuning that will allow the prediction of the observation probability associated with each HMM states.
2. Convolutional neural networks, like in neural networks, learnable kernels (filters) receive the audio spectral features as input, over which a weighted sum is computed and passed through an activation function. A CNN is typically made of a series of convolutional layers on top of which pooling layers are added for the purpose of down-sampling the learned feature maps. These layers are usually followed by one or more dense layers (see Figure 9). A fully convolutional network (FCN) is a variant of CNN extended with fully connected layers. For feature extraction, we use a logarithmic (log)-scaled mel-spectrogram with an appropriate number of components (bands) covering the audible frequency range of a cough (0–22,050 Hz), using a window size and a hop size of the same duration based on the number of samples. As mentioned earlier in this paper, the conversion to log-scaled Mel takes into account that human ear responds better to sounds by perceiving its logarithmic changes in intensity, thus the rational for using the decibel scale.
3. Recurrent Neural Networks (RNNs) allow cyclical connections in a feed-forward neural networks, which allows them to incorporate contextual information from previous input, and remember past input values in the layers' internal state. This property makes RNNs an attractive choice for sequence to sequence learning. Compared to CNNs, RNNs adopt a different approach for representing he temporal features of information. At each time-step, RNNs compute the output based on the current input and the result of the hidden state at the previous time-step. By doing so, RNNs embed in their processing the temporal dependency of the inputs across the time-steps of the past. That is for bidirectional RNNs, the same process can be employed in reversed order by extending the receptive field into the future. Long short-term memory networks (LSTMs) are a variant of RNNs that exploit the contextual information over longer time intervals to map the input sequence to the output. LSTMs known to be efficient at learning temporal dependencies, and they are applied in a variety of areas, such as such speech recognition and synthesis. They are also used with some success when combined with CNN as front-end (CRNN), for video classification. Although, the applicability of LSTM for sound classification has not been fully investigated, they can be very beneficial given the temporal properties embedded in a cough sound.
4. Integrating fuzzy inference systems into deep learning networks is one of hybrid classification approaches that have proven to be effective methods for making highly accurate predictions from complex data sources in the fuzzy logic domain (i.e., fuzzy sets, fuzzy rules). This combination aims to model vague notions with rigorous mathematical tools and rejects the principle of bivalence for pattern recognition, classification, regression or density estimation. Based on this aspect, many methods can be produced, such as combining convolutional neural network with fuzzy logic which called the Fuzzy Convolutional Neural Network (FCNN). Both neural networks and fuzzy systems have no mathematical model necessary. Neural networks are able to learn from scratch using several learning algorithms but fuzzy systems are based on *a priori* knowledge and not capable to learn. Neural networks based on black-box behavior but fuzzy systems are based on simple interpretation and implementation. Therefore, combining both approaches aims to unite advantages and exclude disadvantages.

### 3.3. Cloud Hosting Solutions

Cloud is important for storing collected raw data and/or useful information using internet of medical thing (54) to improve the decision-making of the physicians and/or monitor the quarantined patients through the proposed digital device. The main benefit of this approach is to extend the use of our proposed system to facilities that people with respiratory illnesses to send their health data and early diagnosis to medical experts with high confidentiality.

## 4. Conclusions

The architectural design of the proposed theoretical framework is composed of two major components. The first one consists of devising a process of collecting the data related to the vital signs of the patient and the recorded cough sound, breathing, and so on. The second part consists of building an integrated hardware/software system for end-users intended to capture a patient's vital signs along with the sound of the coughing. The hardware part of this system includes all the sensing devices whereas the software part contains the data processing as well as the prediction algorithms, which predicts the type of the respiratory illness including COVID-19. The prediction algorithm relies on the sate of the art AI model available in the respiratory illness database in the cloud. For the proposed system to gain traction in the market and be adopted by the public and healthcare systems, a number of KPIs need to be achieved and maintained, including efficient use of our proposed system, effectiveness and timeliness of early diagnosis, diagnosis costs, patient wait time, data transparency, decision-making errors of the used classifier, and patient safety and satisfaction. However, identifying the right KPIs, track them, and organize them in a logical, coherent and useful way, is critical step to achieve a good system evaluation, especially for developing medical devices. To evaluate this kind of systems, we shall conduct a pilot study and/or recruit volunteers (beta testers) to test the usability of the device and its software. Many other quality aspects, such as reliability and performance efficiency can be assessed in the of evaluation process, where the involvement of medical experts will form a crucial role in this process.

The effective and efficient use of our proposed theoretical system is based on the collection of a combination of relevant health data for creating a feature vector to differentiate between multi-class respiratory illnesses. A timely diagnosis is a critical criteria for evaluating such a system; using advanced deep learning methods and dimensionality reduction lead for big data analysis will minimize the classification and diagnosis time. The proposed system is designed to be a portable and low cost diagnostic tool, supported with AI. Many countries in the world have limited resources, where this lab-free device will be handy. In addition, this system will increase data transparency between the patients and the physicians, and the ability to share data and diagnosis by the proposed system, with the medical experts via the cloud would improve timely intervention and reduce any false-positives of false-negatives. Advanced machine learning algorithms with public large-scale datasets are the key to speed up the realization of the proposed system for understanding the relationship between respiratory conditions and coughing patterns.

## Data Availability Statement

The original contributions presented in the study are included in the article/supplementary material, further inquiries can be directed to the corresponding author/s.

## Author Contributions

AB conceived the topic and conducted the conceptual design for the hypothesis and theory. All authors listed have made a substantial, direct and intellectual contribution to the work, and approved it for publication.

## Conflict of Interest

The authors declare that the research was conducted in the absence of any commercial or financial relationships that could be construed as a potential conflict of interest.
